# RNA 3D structure modeling by fragment assembly with small-angle X-ray scattering restraints

**DOI:** 10.1093/bioinformatics/btad527

**Published:** 2023-08-30

**Authors:** Grzegorz Chojnowski, Rafał Zaborowski, Marcin Magnus, Sunandan Mukherjee, Janusz M Bujnicki

**Affiliations:** International Institute of Molecular and Cell Biology, Warsaw 02-109, Poland; European Molecular Biology Laboratory, Hamburg Unit, Hamburg 22607, Germany; International Institute of Molecular and Cell Biology, Warsaw 02-109, Poland; ReMedy International Research Agenda Unit, IMol Polish Academy of Sciences, Warsaw, Poland; International Institute of Molecular and Cell Biology, Warsaw 02-109, Poland; International Institute of Molecular and Cell Biology, Warsaw 02-109, Poland

## Abstract

**Summary:**

Structure determination is a key step in the functional characterization of many non-coding RNA molecules. High-resolution RNA 3D structure determination efforts, however, are not keeping up with the pace of discovery of new non-coding RNA sequences. This increases the importance of computational approaches and low-resolution experimental data, such as from the small-angle X-ray scattering experiments. We present RNA Masonry, a computer program and a web service for a fully automated modeling of RNA 3D structures. It assemblies RNA fragments into geometrically plausible models that meet user-provided secondary structure constraints, restraints on tertiary contacts, and small-angle X-ray scattering data. We illustrate the method description with detailed benchmarks and its application to structural studies of viral RNAs with SAXS restraints.

**Availability and implementation:**

The program web server is available at http://iimcb.genesilico.pl/rnamasonry. The source code is available at https://gitlab.com/gchojnowski/rnamasonry.

## 1 Introduction

Non-coding RNAs (ncRNAs) are involved in the regulation of many cellular processes. New families of ncRNAs are being continuously discovered (Kalvari *et al.* 2021). The function of newly characterized ncRNAs can be deciphered from their structure in an approach that proved very successful for proteins (Miao *et al.* 2020). The experimental determination of high-resolution 3D structures of ncRNAs (e.g. by X-ray crystallography, nuclear magnetic resonance, or cryo-electron microscopy) is however difficult, and only a very small fraction of ncRNA families have high-resolution structures available for at least one member (134 out of 4108 according to Rfam 14.9 (Kalvari *et al.* 2021)). Computational structure prediction methods offer an alternative to experimental structure determination, but the purely theoretical models are often of limited accuracy. One promising approach is the computational modeling with the use of restraints derived from low-resolution experimental data (Bernetti *et al.* 2021, Mazzanti *et al.* 2021, Fang *et al.* 2022). To address this issue, we developed RNA Masonry, a computer program for fully automated modeling of RNA molecules by assembly of recurrent 3D motifs, with the use of low-resolution restraints. The 3D motifs (further referred to as fragments) are retrieved from the RNA Bricks database (Chojnowski *et al.* 2014), which catalogues recurrent substructures observed in experimentally determined, high-resolution RNA structural models available in the Protein Data Bank (wwPDB Consortium 2018).

RNA Masonry provides two basic functionalities: (i) *de novo* fragment assembly and (ii) RNA model refinement. The input is either RNA sequence with the secondary structure or a preliminary RNA 3D structural model. The input atomic model is analyzed by the ClaRNA program (Walen *et al.* 2014) to produce the secondary structure, which is then used as input to RNA Masonry in the *de novo* modeling mode. Pseudo-knots in the secondary structure are removed and used as additional contact restraints.

RNA Masonry is based on hierarchical organization of RNA molecules, which are composed of 3D motifs defined at a secondary structure level; double-stranded helices, single-stranded fragments, and various types of loops. During the model assembly, the motifs are used as a whole, which strictly preserves the input secondary structure. The program accepts restraints for long-range tertiary interactions. Additionally, the model building can be restrained with a goodness-of-fit to the experimental small-angle X-ray scattering (SAXS) data, which is calculated using FoXS (Schneidman-Duhovny *et al.* 2016) or CRYSOL (Franke *et al.* 2017) depending on user preferences.

There are other methods available that can assemble RNA 3D structures from fragments of experimentally determined structures like Assemble (Jossinet *et al.* 2010) and RNAComposer (Popenda *et al.* 2012). There are also approaches designed specifically for the purpose of modeling with SAXS restraints (Gajda *et al.* 2013), or filtering large sets of tentative models (Yang *et al.* 2010). To the best of our knowledge, however, RNA Masonry is the only tool that combines statistical potential with experimental restraints and uses a regularly updated database of fragments (RNA Bricks is updated weekly, http://genesilico.pl/rnabricks2).

## 2 Materials and methods

### 2.1 Input processing

If the input is an RNA atomic model (mmCIF or PDB format), the program annotates its secondary structure with ClaRNA which is further processed analogously to a secondary structure given directly on input for *de novo* modeling.

Pseudo-knots are removed from the input secondary structure using the K2N library (Smit *et al.* 2008) to maximize the number of nested base pairs (Ponty 2006), and stored as additional restraints. The pseudo-knot free secondary structure is subsequently decomposed into structural motifs and encoded as an RNA motif-graph introduced in the RNA Bricks database as described previously (Chojnowski *et al.* 2014). Briefly, the graph nodes correspond to RNA structural motifs (double-stranded helices, loops, and single-stranded fragments). The graph edges denote nucleotides shared by two neighboring motifs. Owing to the absence of pseudo-knots in the basic data structure, the motif graphs are trees, albeit not necessarily rooted. Pseudo-knots are nonetheless enforced at the level of structural restraints.

### 2.2 Selecting RNA 3D fragments

After building the motif-graph, which is a basic data structure used in RNA Masonry, 3D fragments from RNA Bricks database are assigned to each of its nodes. A fragment assigned to a node must match its secondary structure (stem or a loop with specific topology), but no sequence information is considered. If the number of fragments assigned to a node is fewer than a user-defined threshold (10 by default) additional fragments are generated by circular permutations (e.g. by changing ends of symmetrical, internal loops). If needed, new fragments are also automatically built from smaller ones by introducing insertions using ModeRNA (Rother *et al.* 2011). Finally, each fragment is mutated to the target sequence. Any steric conflicts, unsatisfied pseudo-knot restraints, or implausible geometries introduced at this stage are ignored, but penalized later during fragment assembly steps by the SimRNA (Boniecki *et al.* 2016) scoring function.

### 2.3 Starting RNA model assembly

If RNA 3D structure is given as an input, it is used as a starting model without any further modifications. Otherwise, the program assembles a starting model with a possibly small number of severe clashes, which increases the convergence of the subsequent refinement step.

In principle, the aim is to find a configuration of fragments, assigned to each of the motif-graph nodes, which minimizes the number of clashes in a complete model. A clash occurs when two glycosidic nitrogens (N1 for pyrimidines, N9 for purines) are closer than 6 Å apart. To reduce the combinatorial complexity of the task, the assembly process is initiated from the terminal elements of the structure (the motif graph ‘leaves’) and proceeds iteratively by adding a single node at a time. At each step, from all possible partial models only statistically relevant representatives are selected as described by Hajdin *et al.* (2010).

### 2.4 Model optimization

The starting model is optimized in a replica exchange Monte-Carlo simulation with a geometric distribution of temperature levels. We use a scoring function based on the SimRNA statistical potential (Boniecki *et al.* 2016) that enables the use of the long-range tertiary contact restraints (e.g. to indicate pseudo-knots). If SAXS curve is given on input the score is additionally multiplied by a correction factor penalizing poor agreement of a full-atom model representation with experimental data. The correction factor quantifies deviations of the χ^2^ value from 1 with a mixture of two Gaussians with variances 1 and 10, where a perfect fit (χ^2^ of 1) corresponds to a value 1.5.

In a single step, a random motif-graph node is selected. Next, from a set of fragments assigned to the node a random one is selected and inserted into corresponding position in the model. For all motifs except terminal loops, this operation changes conformation of a whole domain in the RNA model. The user-provided secondary structure is preserved at each step. The best-scored (lowest pseudo-energy) model constructed during the entire simulation is returned to the user.

## 3 Structural studies of viral RNAs with SAXS restraints

**Figure 1.**
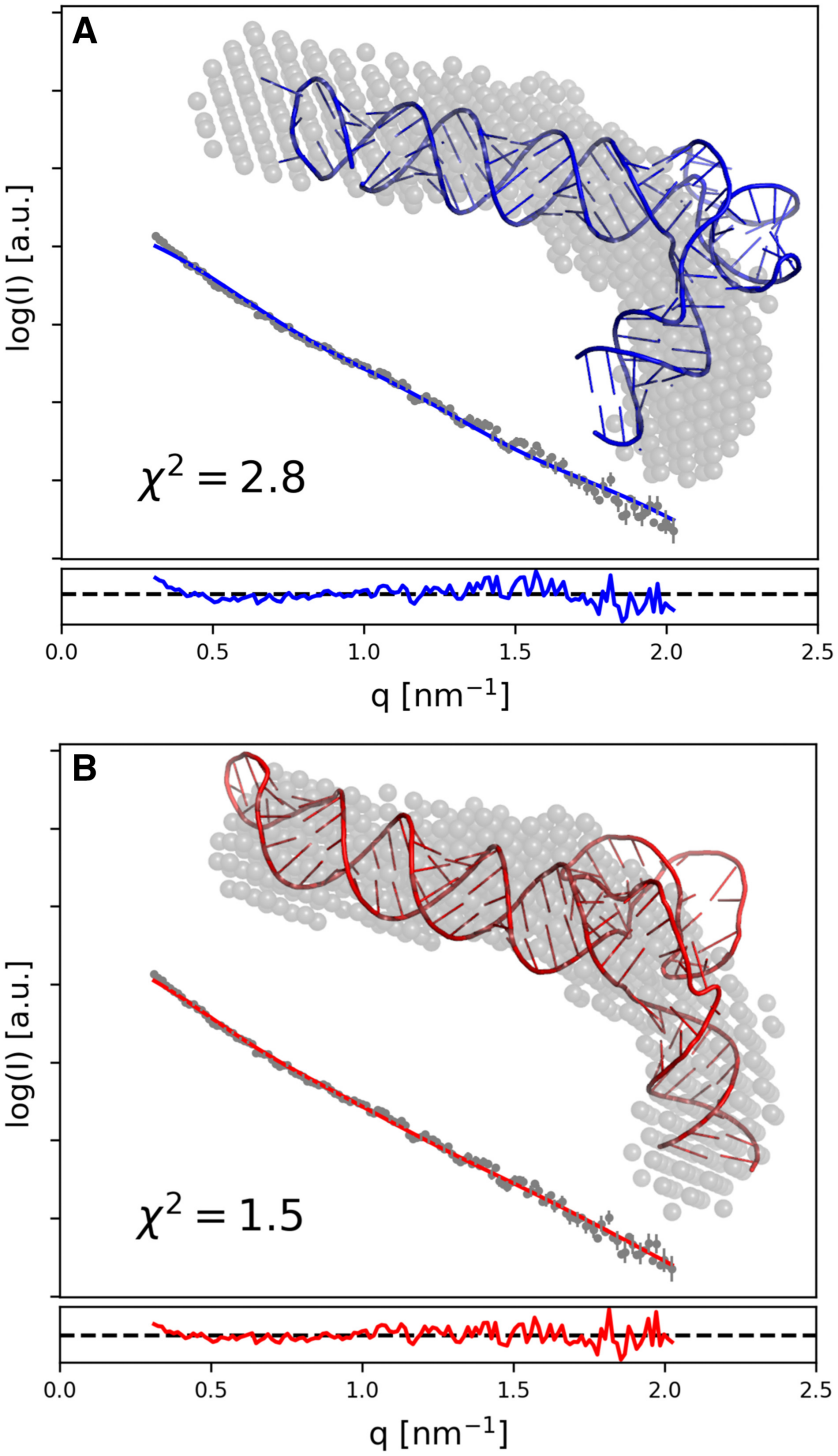
Crystal structure of adenovirus virus-associated RNA (A) was observed to have a conformation inconsistent with results of earlier SAXS experiments. RNA Masonry was used to optimize the fit to experimental SAXS data preserving the crystal structure secondary contacts (B). An *ab initio* model obtained with DAMMIF isshown with grey spheres. Residuals of model-calculated and experimental SAXS curve fit are shown in the bottom panels.

We used an early version RNA Masonry to model the adenovirus virus-associated RNA (VA-I) structure with SAXS restraints (Dzananovic *et al.* 2017). Subsequently, a crystal structure of VA-I has been determined and turned out to be inconsistent with the results of scattering experiments with goodness-of-fit parameter (χ^2^) estimated using CRYSOL of 2.8 (Hood *et al.* 2019) (Fig. 1A). We used RNA Masonry to optimize the fit of the structure to the SAXS curve, while preserving its secondary structure, which could have been very accurately determined using a crystal structure model. The resulting model fits better not only to the experimental data (χ^2^ = 1.5), but also to the *ab initio* reconstruction obtained independently using DAMMIF (Franke and Svergun 2009) (Fig. 1B). This result supports a hypothesis that in solution and in the absence of crystal-lattice constraints VA-I may be flexible and sample additional conformations, possibly at the expense of the pseudo-knot disruption (Hood *et al.* 2019).

## 4 Implementation

RNA Masonry and all utility programs were implemented in Python 3 and C++ with an extensive use of routines from the Computational Crystallography Toolbox (cctbx) (Grosse-Kunstleve *et al.* 2002), Numpy, Scipy, NetworkX, Biopython (Cock *et al.* 2009), ClaRNA, ModeRNA, and K2N libraries. The web server engine used in this work is a part of rna-tools toolbox (Magnus *et al.* 2020) and can be freely reused for new applications.

## 5 Conclusions

RNA Masonry is a computer program for fully automated modeling of RNA 3D structures by fragment assembly. The RNA structure representation used here significantly reduces the number of degrees of freedom, which in principle equals the number of joints (internal loops) in the target structure. The small number of degrees of freedom significantly reduces the modeling time (e.g. compared to SimRNA simulations). In the case of modeling with SAXS data it also reduces the risk of overfitting. The SAXS experiments provide a number of unique observations much smaller than the already limited number of experimental data points (Konarev and Svergun 2015). This increases the impact of experimental errors on models with more parameters. However, in cases where SAXS curves represent an average over multiple model states, a small number of RNA Masonry model parameters may be not enough to explain the complexity of experimental data. Overall, we show using simulated and experimental SAXS data that RNA Masonry can provide accurate models in most of the cases (see Supplementary data).

It must be stressed, that the presented approach may be unable to model fine details of RNA structures (e.g. non-canonical interactions) that are not encoded within the 3D motifs used for assembly. These, however, can be modeled using complementary high-resolution approaches (e.g. using QRNAS (Stasiewicz *et al.* 2019), SimRNA, or ROSETTA/FARFAR (Das *et al.* 2010)). This issue does not affect alternative approach where SAXS data are used for filtering large decoy sets generated using methods that can explicitly model non-canonical interactions, for example MC-Sym and MC-Fold pipeline (Yang *et al.* 2010).

The current RNA Masonry version selects RNA fragments for model building based exclusively on canonical secondary structure constraints. It was observed, however, that the presence of certain motif types and coaxial stacking between adjacent helices can be reliably predicted from sequence (Tyagi and Mathews 2007, Cruz and Westhof 2011). Both these *a priori* information could be in principle used during model building in RNA Masonry to increase reliability of resulting models. This can be achieved by a more restrictive selection of fragments for model assembly and penalizing the absence of expected stacking interactions by the SimRNA scoring function. We plan such an extension in further releases of the software.

## Supplementary Material

btad527_Supplementary_Data

## Data Availability

The data underlying this article are available in the online supplementary material.
