# Supplementary material for: RNA 3D structure modeling by fragment assembly with small-angle X-ray scattering restraints
Source: Bioinformatics. 2023 Aug 30;39(9):btad527. doi: 10.1093/bioinformatics/btad527 (PMC10474949; doi:10.1093/bioinformatics/btad527)
Supplement: btad527_Supplementary_Data [file btad527_supplementary_data.pdf]

RNA 3D structure modeling by fragment assembly with Small Angle X-ray Scattering restraints

Grzegorz Chojnowski[1,2,†,*], Rafał Zaborowski[1,%,†], Marcin Magnus[3,+], Sunandan Mukherjee[1], and Janusz M. Bujnicki[1]

[1]International Institute of Molecular and Cell Biology, Trojdena 4, 02-109 Warsaw, Poland
[2]European Molecular Biology Laboratory, Hamburg Unit, Notkestrasse 85, 22607 Hamburg, Germany
[3] ReMedy International Research Agenda Unit, IMol Polish Academy of Sciences, Warsaw, Poland
[%]Present address: Faculty of Mathematics, Informatics, and Mechanics, University of Warsaw, Banacha 2, 02-097 Warsaw, Poland
[+] Present address: Department of Molecular and Cellular Biology, Harvard University, Cambridge, USA
[†]These authors contributed equally to this work
*To whom correspondence should be addressed.

RNA Masonry benchmarks with secondary structure and Small Angle X-ray Scattering restraints

To evaluate the accuracy of RNA Masonry, we selected eight RNA structures from the Protein Data Bank (PDB) (Berman *et al.*, 2000) as a benchmark dataset (Table 1). Additionally, we modelled structures of two RNA molecules with scattering data downloaded from SASBDB (Kikhney *et al.* 2020).

1. Results evaluation criteria

Since RNA Masonry uses the statistical potential of SimRNA (Boniecki *et al.*, 2016), we performed structure prediction with SimRNAweb (Magnus *et al.*, 2016) using sequences with restraints on secondary structure, and considered them as a control to understand the effect of SAXS curves in the prediction. RNA Masonry runs were performed with secondary structure and SAXS curve restraints. Secondary structures used in all the simulations were extracted from the native structures using ClaRNA (Waleń *et al.*, 2014). We used rna-tools (Magnus *et al.*, 2020) to calculate all atoms' root mean square deviations (RMSD), CRYSOL (Svergun *et al.*, 1995) for calculating χ^2 values, and ClaRNA (Waleń *et al.*, 2014) for calculating the canonical and non-canonical contacts used for calculating Matthews correlation coefficient (MCC) with 1D2DSimScore (Moafinejad *et al.*, 2023) using the following equation:

$$MCC = \frac{TP \times TN - FP \times FN}{\sqrt{(TP+FP)(TP+FN)(TN+FP)(TN+FN)}} \qquad (1)$$

2. Simulated Small Angle X-ray Scattering data

Simulated SAXS patterns were created using the atomic coordinates of these eight structures with the IMSIM tool (Franke *et al.*, 2020) from the ATSAS package (Manalastas-Cantos *et al.*, 2021). We generated SAXS patterns at sample concentrations (c) c=0.5 mg/ml, c=2.0 mg/ml, and c=8.0 mg/ml.

RNA molecule	PDB id	Sequence length	Resolution (Å)
Viral RNA pseudoknot	1l2x	28	1.25
Rigorously conserved RNA element within the SARS virus genome	1xjr	47	2.7
Alu domain of the mammalian SRP RNA	1e8o	50	3.2
Unmodified tRNAPhe from Escherichia coli	3l0u	76	3.0
Glycine riboswitch (unbound state)	3ox0	88	3.049
Escherichia coli yybP-ykoY Mn riboswitch in the Mn2+-free state	4y1m	108	3
Speciation of a group I intron into a lariat capping ribozyme	4p8z	188	3.85
Structure of the Tetrahymena Ribozyme with base triple sandwich	1x8w	242	3.8

Supplementary table 1. List of RNA structures used for benchmark analysis of RNA Masonry.

3. Performance of RNA Masonry with simulated datasets

We assessed the performance of RNA Masonry using three different criteria. We used all atoms root-means-square deviation (RMSD) with respect to the native structure to quantify the overall structural dissimilarities in 3D space, χ^2 values to evaluate the agreement of the predicted model with the scattering data, and MCC using the native structure as a reference to estimate the prediction accuracy of canonical and non-canonical contacts along with stacking interactions (please refer to supplementary table 2). The average RMSD for all eight structures in the benchmark set shows better prediction accuracy by RNA Masonry (average RMSD = 9.43 Å) compared to the

SimRNA web server (average RMDS = 14.85 Å). Out of eight cases, RNA Masonry performed better for five cases (PDB ids: 1e8o, 1xjr, 3l0u, 3ox0, and 4y1m, Supplementary figure 1), and comparable accuracies in two cases (PDB ids: 1l2x, 4p8z). In the case of 4p8z, the overall prediction accuracies of both RNA Masonry and SimRNA are poor with RMSD over 20 Å. We could not compare the results of *Tetrahymena* ribozyme with the base triple sandwich (PDB id: 1x8w) as the sequence length of the molecule is beyond the acceptable range of the SimRNA web server (which is 200 nts). All the inputs (sequences, secondary structures, and the simulated SAXS curves) and the predicted models are available at a Zenodo repository (https://doi.org/10.5281/zenodo.8108714).

	χ2			RMSD	MCC
	c=0.5	c=2.0	c=8.0		
**1e8o**					
SimRNA-Web Cluster01	1.11	2.55	17.26	6.46	0.90
SimRNA-Web Cluster02	1.22	3.78	30.85	9.56	0.90
SimRNA-Web Cluster03	1.13	2.85	18.86	4.43	0.95
SimRNA-Web Cluster04	1.46	6.55	55.38	15.37	0.80
SimRNA-Web Cluster05	1.76	9.89	80.42	15.87	0.83
rms_C0.5	1.015			2.94	0.917
rms_C2.0		1.432		3.92	0.917
rms_C8.0			3.372	5.40	0.89
native	1.02	1.362	5.616	0	1.00
**1l2x**					
SimRNA-Web Cluster01	1.07	1.1	2.15	3.81	1.00
SimRNA-Web Cluster02	1.06	1.13	2.72	4.12	0.89
SimRNA-Web Cluster03	1.11	1.48	5.67	3.98	0.942
SimRNA-Web Cluster04	1.09	1.23	3.62	3.73	0.89
SimRNA-Web Cluster05	1.12	1.67	8.75	4.48	0.94
rms_C0.5	1.151			11.40	0.79
rms_C2.0		3.094		3.19	0.865
rms_C8.0			25.28	10.00	0.79
native	1.061	0.99	1.209	0	1.00
**1xjr**					
SimRNA-Web Cluster01	1.61	7.9	72.12	9.89	0.689
SimRNA-Web Cluster02	1.31	4.38	35.17	8.17	0.71
SimRNA-Web Cluster03	1.82	9.85	76.61	11.49	0.67
SimRNA-Web Cluster04	1.73	8.73	69.82	8.54	0.64
SimRNA-Web Cluster05	2.34	16.38	144.39	9.86	0.732
rms_C0.5	1.522			4.40	1.00
rms_C2.0		2.354		5.47	1.00
rms_C8.0			1.695	10.24	1.00
native	1.027	0.9999	1.239	0	1.00
**3l0u**					
SimRNA-Web Cluster01	1.8	7.84	49.93	19.88	0.798
SimRNA-Web Cluster02	1.34	4.43	25.53	13.07	0.852
SimRNA-Web Cluster03	2.09	10.63	70.89	21.05	0.81
SimRNA-Web Cluster04	2.06	11.78	93.06	18.92	0.834

SimRNA-Web Cluster05	1.55	5.55	33.12	23.54	0.786
rms_C0.5	1.015			4.08	1.00
rms_C2.0		1.432		4.60	1.00
rms_C8.0			3.372	6.67	1.00
native	1.01	1.025	1.343	0	1.00
**3ox0**					
SimRNA-Web Cluster01	1.82	8.15	41.78	16.78	0.88
SimRNA-Web Cluster02	1.21	3.85	24.95	12.08	0.84
SimRNA-Web Cluster03	1.25	3.16	14.38	16.08	0.95
SimRNA-Web Cluster04	1.49	5.01	24.88	18.66	0.89
SimRNA-Web Cluster05	1.14	2.65	13.36	11.72	0.946
rms_C0.5	1.395			8.85	1.00
rms_C2.0		1.179		4.55	1.00
rms_C8.0			1.497	4.74	1.00
native	0.9648	1.069	1.62	0	1.00
**4p8z**					
SimRNA-Web Cluster01	1.6	6.2	38.39	20.36	0.90
SimRNA-Web Cluster02	1.66	8.64	66.55	27.24	0.86
SimRNA-Web Cluster03	1.44	6.21	51.85	30.17	0.85
SimRNA-Web Cluster04	1.97	12.31	93.62	27.17	0.92
SimRNA-Web Cluster05	1.82	10.21	77.25	19.63	0.92
rms_C0.5	3.092			21.62	0.921
rms_C2.0		5.569		29.73	0.874
rms_C8.0			88.12	26.81	0.902
native	1.05	1.124	1.859	0	1.00
**4y1m**					
SimRNA-Web Cluster01	1.24	3.91	20.17	18.36	-0.002
SimRNA-Web Cluster02	1.66	6.7	35.86	27	-0.002
SimRNA-Web Cluster03	1.1	2.63	13.75	12.72	-0.002
SimRNA-Web Cluster04	1.32	3.77	18.18	25.73	-0.002
SimRNA-Web Cluster05	1.63	7.49	51.58	19.9	-0.002
rms_C0.5	1.072			6.01	1.00
rms_C2.0		1.21		17.60	1.00
rms_C8.0			2.267	5.88	1.00
native	0.9676	1.14	2.205	0	1.00
**1x8w**					
rms_C0.5	1.153			24.43	0.936
rms_C2.0		150.2		43.77	0.93
rms_C8.0			394.5	35.54	0.936
native	0.9703	1.130	1.885	0	1.00

Supplementary table 2: χ2, RMSD, and MCC values for the models predicted by SimRNAweb (without SAXS data), RNA Masonry (with SAXS data), and the experimentally determined reference models.

RNA Masonry showed a remarkable average MCC value of 0.95 (compared to an average MCC = 0.73 for models predicted by the SimRNA web server), with a distribution ranging from 0.79 to 1.00. In two cases with large RNA structures (PDB id: 4p8z and 1x8w), RNA Masonry performed poorly in terms of RMSDs. These models achieved an MCC of 0.87 or better, but the χ^2 values indicate poor agreement with the

SAXS pattern. In most of the cases, RNA Masonry was able to achieve the best fitting with c=0.5 (average χ^2=1.46), followed by c=2.0 (average χ^2=2.32), and c=8.0 (average χ^2=6.25).

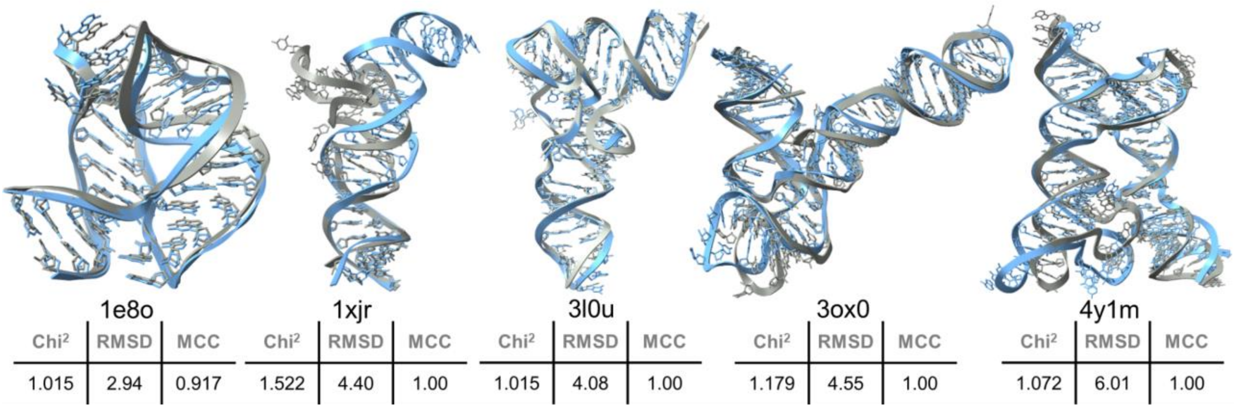

	1e8o			1xjr			3l0u			3ox0			4y1m	
Chi²	RMSD	MCC	Chi²	RMSD	MCC	Chi²	RMSD	MCC	Chi²	RMSD	MCC	Chi²	RMSD	MCC
1.015	2.94	0.917	1.522	4.40	1.00	1.015	4.08	1.00	1.179	4.55	1.00	1.072	6.01	1.00

Supplementary figure 1: Experimentally determined models from PDB (in grey) superposed with the models predicted by RNA Masonry using simulated SAXS curve.

4. Performance of RNA Masonry with experimental data

Additionally, we modelled structures of two RNA molecules with scattering data downloaded from SASBDB: truncated P5abc subdomain of Tetrahymena ribozyme (tp5abc; SAXDB id: SASDCK4), and Xrn1 resistance RNA2 from Zika virus (ZIKV-xrRNA2; SAXDB id: SASDGF3). We did homology modelling of tp5abc using the crystal structure of group I ribozyme domain (PDB id: 1gid) as a template. For ZIKV-xrRNA2, we used the crystal structure of exonuclease-resistant RNA from the Zika virus (PDB id: 5tpy) as a template for homology modelling. In both cases, ModeRNA (Rother *et al.*, 2011) was used for homology modelling. We used two different modes: *de novo* prediction and refinement of the homology models with help of the SAXS curve. For tp5abc and ZIKV-xrRNA2, RNA Masonry achieved χ^2 values of 3.70 and 0.80, respectively, while predicting in *de novo* mode with respective sequences, secondary structures of the homologous templates, and SAXS curves as inputs (Supplementary figure 2). Whereas, after refinement, the χ^2 values of the refined models of tp5abc and ZIKV-xrRNA2 are 2.82 and 4.64, respectively. The RNA Masonry prediction with the SAXS curve of ZIKV-xrRNA2 achieved better χ^2 than the crystal structure reported in the study (χ^2 = 76.08).

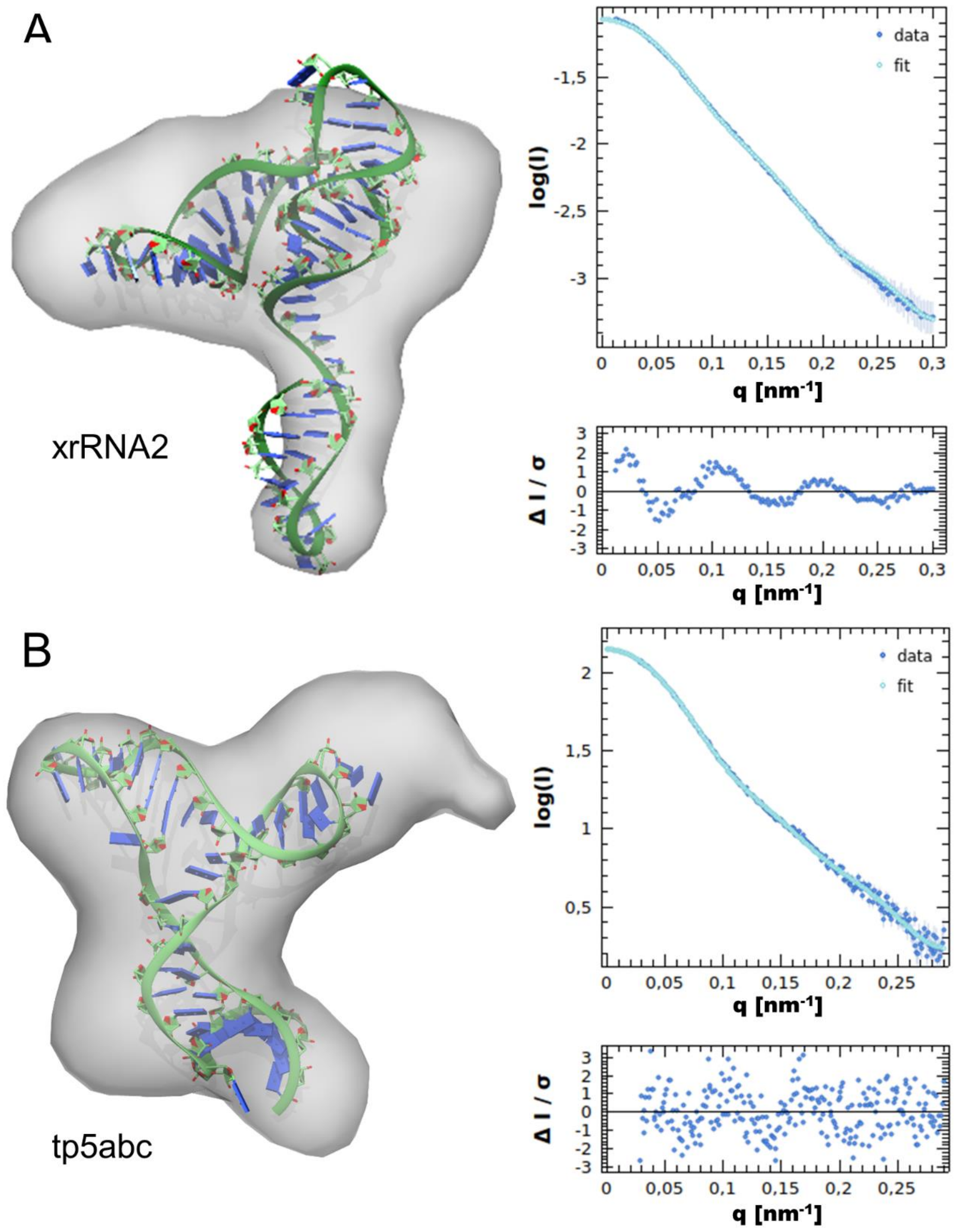

Supplementary figure 2: Fit of the models predicted by RNA Masonry against the dummy atom model and the SAXS curve for (A) xrRNA2 resistance RNA2 from Zika virus and (B) truncated tp5abc subdomain of Tetrahymena ribozyme.

5. Execution time

The execution time of RNA Masonry depends on the sequence length of the RNA. We reported the execution times of five of the RNA molecules from our benchmark dataset of various sequence lengths (ranging from 47 to 188 nucleotides), and two different sample concentrations (0.5 and 2.0). RNA Masonry predictions were performed on a modern laptop computer with AMD Ryzen 9 @ 3.3 GHz processor and 64-bit Linux

operating system (Ubuntu 20.04). We observed that the time required by RNA Masonry directly proportional to the size (sequence length) of the RNA molecules (Supplementary figure 3).

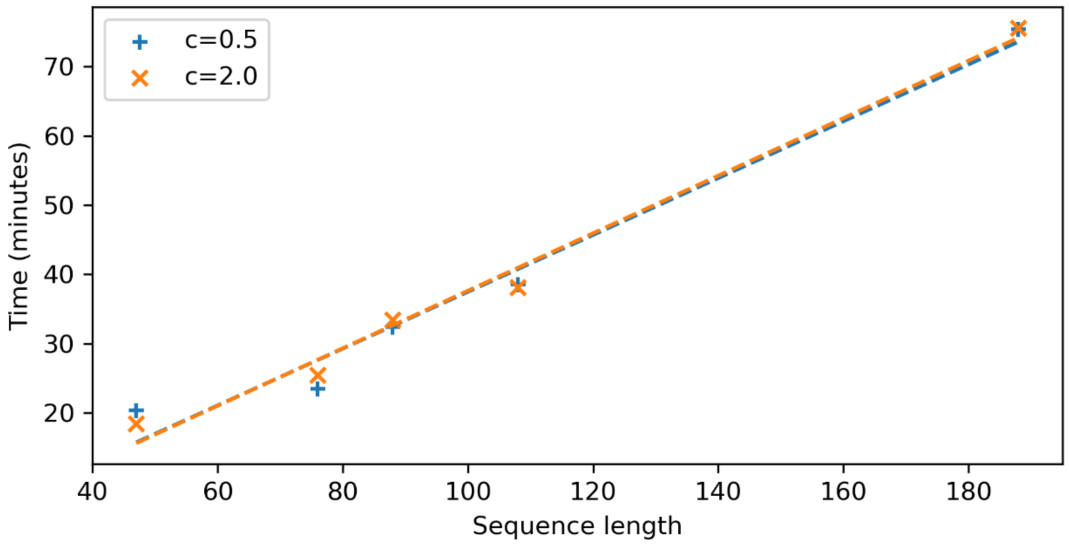

Supplementary figure 3: Sequence length vs. execution time required by RNA Masonry

List of commands used in RNA Masonry benchmark study

```
rnamasonry -i 1e8o.seq --saxs-data 1e8o00_norm_c0.5.dat --steps 50
rnamasonry -i 1e8o.seq --saxs-data 1e8o00_norm_c2.0.dat --steps 50
rnamasonry -i 1e8o.seq --saxs-data 1e8o00_norm_c8.0.dat --steps 50

rnamasonry -i 1l2x.seq --saxs-data 1l2x00_norm_c0.5.dat --steps 50
rnamasonry -i 1l2x.seq --saxs-data 1l2x00_norm_c2.0.dat --steps 50
rnamasonry -i 1l2x.seq --saxs-data 1l2x00_norm_c8.0.dat --steps 50

rnamasonry -i 1xjr.seq --saxs-data 1xjr00_norm_c0.5.dat --steps 50
rnamasonry -i 1xjr.seq --saxs-data 1xjr00_norm_c2.0.dat --steps 50
rnamasonry -i 1xjr.seq --saxs-data 1xjr00_norm_c8.0.dat --steps 50

rnamasonry -i 3l0u.seq --saxs-data 3l0u00_norm_c0.5.dat --steps 50
rnamasonry -i 3l0u.seq --saxs-data 3l0u00_norm_c2.0.dat --steps 50
rnamasonry -i 3l0u.seq --saxs-data 3l0u00_norm_c8.0.dat --steps 50

rnamasonry -i 3ox0.seq --saxs-data 3ox000_norm_c0.5.dat --steps 50
rnamasonry -i 3ox0.seq --saxs-data 3ox000_norm_c2.0.dat --steps 50
rnamasonry -i 3ox0.seq --saxs-data 3ox000_norm_c8.0.dat --steps 50

rnamasonry -i 4p8z.seq --saxs-data 4p8z00_norm_c0.5.dat --steps 50
rnamasonry -i 4p8z.seq --saxs-data 4p8z00_norm_c2.0.dat --steps 50
rnamasonry -i 4p8z.seq --saxs-data 4p8z00_norm_c8.0.dat --steps 50
```

```
rnamasonry -i 4y1m.seq --saxs-data 4y1m00_norm_c0.5.dat --steps 50
rnamasonry -i 4y1m.seq --saxs-data 4y1m00_norm_c2.0.dat --steps 50
rnamasonry -i 4y1m.seq --saxs-data 4y1m00_norm_c8.0.dat --steps 50

rnamasonry -i 1x8w.seq --saxs-data 1x8w00_norm_c0.5.dat --steps 50
rnamasonry -i 1x8w.seq --saxs-data 1x8w00_norm_c2.0.dat --steps 50
rnamasonry -i 1x8w.seq --saxs-data 1x8w00_norm_c8.0.dat --steps 50

rnamasonry -i tp5abc.seq --saxs-data SASDCK4.dat --steps 50
rnamasonry -i ZIKV-x2rRNA.seq --saxs-data SASDGF3.dat --steps 50
```

References

Berman,H.M. *et al.* (2000) The Protein Data Bank. *Nucleic Acids Res.*, **28**, 235–242.

Boniecki,M.J. *et al.* (2016) SimRNA: a coarse-grained method for RNA folding simulations and 3D structure prediction. *Nucleic Acids Res.*, **44**, e63.

Franke,D. *et al.* (2020) Simulation of small-angle X-ray scattering data of biological macromolecules in solution. *J. Appl. Crystallogr.*, **53**, 536–539.

Kikhney, Alexey G., et al. (2020) SASBDB: Towards an automatically curated and validated repository for biological scattering data. *Protein Science* 29.1, 66-75.

Magnus,M. *et al.* RNA-Puzzles toolkit: a computational resource of RNA 3D structure benchmark datasets, structure manipulation, and evaluation tools. *Nucleic Acids Res.*, **48**, 576–588.

Magnus,M. *et al.* (2016) SimRNAweb: a web server for RNA 3D structure modeling with optional restraints. *Nucleic Acids Res.*, **44**, W315–9.

Manalastas-Cantos,K. *et al.* (2021) ATSAS 3.0: expanded functionality and new tools for small-angle scattering data analysis. *Journal of Applied Crystallography*, **54**, 343–355.

Moafinejad,S.N. *et al.* (2023) 1D2DSimScore: A novel method for comparing contacts in biomacromolecules and their complexes. *Protein Science*, **32**.

Rother,M. *et al.* (2011) ModeRNA: a tool for comparative modeling of RNA 3D structure. *Nucleic Acids Res.*, **39**, 4007–4022.

Svergun,D. *et al.* (1995) CRYSOL– a Program to Evaluate X-ray Solution Scattering of Biological Macromolecules from Atomic Coordinates. *Journal of Applied Crystallography*, **28**, 768–773.

Waleń,T. *et al.* (2014) ClaRNA: a classifier of contacts in RNA 3D structures based on a comparative analysis of various classification schemes. *Nucleic Acids Res.*, **42**, e151.
